# Ecology of sleeping: the microbial and arthropod associates of chimpanzee beds

**DOI:** 10.1098/rsos.180382

**Published:** 2018-05-16

**Authors:** Megan S. Thoemmes, Fiona A. Stewart, R. Adriana Hernandez-Aguilar, Matthew A. Bertone, David A. Baltzegar, Russell J. Borski, Naomi Cohen, Kaitlin P. Coyle, Alexander K. Piel, Robert R. Dunn

**Affiliations:** 1 Department of Applied Ecology and Keck Center for Behavioral Biology, North Carolina State University, Raleigh, NC, USA; 2 Department of Entomology and Plant Pathology, North Carolina State University, Raleigh, NC, USA; 3 Department of Biological Sciences, North Carolina State University, Raleigh, NC, USA; 4 Genomic Sciences Laboratory, Office of Research, Innovation and Economic Development, North Carolina State University, Raleigh, NC, USA; 5 Ugalla Primate Project, Katavi Region, Tanzania; 6 School of Natural Sciences and Psychology, Liverpool John Moores University, Liverpool, UK; 7 Department of Archaeology and Anthropology, University of Cambridge, Cambridge, UK; 8 Centre for Ecological and Evolutionary Synthesis, Department of Biosciences, University of Oslo, Oslo, Norway; 9 The Center for Macroecology, Ecology and Conservation, Museum of Natural History, University of Copenhagen, Copenhagen, Denmark

**Keywords:** chimpanzee, nest, bed, microbiome, hygiene hypothesis, built environment

## Abstract

The indoor environment created by the construction of homes and other buildings is often considered to be uniquely different from other environments. It is composed of organisms that are less diverse than those of the outdoors and strongly sourced by, or dependent upon, human bodies. Yet, no one has ever compared the composition of species found in contemporary human homes to that of other structures built by mammals, including those of non-human primates. Here we consider the microbes and arthropods found in chimpanzee beds, relative to the surrounding environment (*n* = 41 and 15 beds, respectively). Based on the study of human homes, we hypothesized that the microbes found in chimpanzee beds would be less diverse than those on nearby branches and leaves and that their beds would be primarily composed of body-associated organisms. However, we found that differences between wet and dry seasons and elevation above sea level explained nearly all of the observed variation in microbial diversity and community structure. While we can identify the presence of a chimpanzee based on the assemblage of bacteria, the dominant signal is that of environmental microbes. We found just four ectoparasitic arthropod specimens, none of which appears to be specialized on chimpanzees or their structures. These results suggest that the life to which chimpanzees are exposed while in their beds is predominately the same as that of the surrounding environment.

## 1. Introduction

Humans modify landforms and build complex networks of structures in which we gather in groups, store goods and protect ourselves from harsh environmental conditions. Since the advent of houses, which occurred between 20 000 [1–4] and 300 000 years ago [5], humans have become increasingly separated from the outdoor environment. Though there is cultural variation in the design and use of buildings globally, human interactions with other organisms now occur primarily within built structures [6]. It has been suggested that changes in the types and diversity of species with which we interact have been to our detriment, whether because we are no longer exposed to the diversity of environmental bacteria necessary for our immune systems to fully develop (e.g. the hygiene hypothesis [7]), or because we fail to acquire commensal species on which our physical health and mental well-being depend. A large body of the literature [8–13], including a number of recent high-profile books [14–17], now considers the idea that these shifts in our interactions with other organisms are making us sick. To varying extents, such work is predicated on the idea that our ancestors were exposed to more and different kinds of microbes than we are currently, whether through various daily activities or while they slept. Yet, to our knowledge no study has compared the species found in human homes, or more generally in the modern built environment, to those found in structures built by other mammals.

Many mammals sleep on the bare ground or in natural cavities, but a subset of mammals constructs modified structures in which to rest. The mammals that build these structures include rodents and other taxa that dig burrows [18–19] and a smaller group of mammals, including some primate species that build modified aboveground sleeping places referred to, variously, as roosts, nests or beds [20–21]. Great apes, including chimpanzees (*Pan troglodytes*), bonobos (*Pan paniscus*), gorillas (*Gorilla* spp.) and orangutans (*Pongo* spp.), all build at least one bed a day to be used for resting before abandonment the following morning [22]. Owing to the pervasiveness of this behaviour and the frequency of bed construction, it has been argued that these beds are the most prevalent form of technology and material culture among extant great apes [23–24]. Although great ape species differ in social organization, behaviour and diet, all construct their beds in a similar manner [22].

Chimpanzee beds, perhaps the best studied of the great ape beds, are complex structures built by interweaving branches into a secure foundation covered by a leafy mattress. These beds have been suggested to provide protection from the wind and other inclement weather, offer refuge from predators and increase comfort while resting. They are also hypothesized to reduce exposure to pests and pathogens [21,24–31]. Chimpanzees spend over half their lives in beds, and they are selective in the materials they use for construction, as well as to where they choose to build them [32–35]. Because chimpanzees spend many hours in their beds each day, these structures are likely to influence which species colonize the skin, guts and other habitats of chimpanzee bodies, and their exposures to such groups are likely to have an impact on their immune systems.

Here we consider the bacteria and arthropods found in chimpanzee beds. More specifically, we consider the diversity and likely origin of such species. Human homes are full of thousands of species that slough off our bodies or consume dead skin, food waste and the house materials themselves [36]. But it has been suggested that what is missing from many homes are the bacteria and other organisms associated with soils, leaves and outdoor habitats [7,8]. Implicitly, this body of research presumes that our ancestors were exposed to microbes and insects from diverse environmental sources, including during the hours in which they slept. We might predict the same for extant non-human great apes, such as chimpanzees. Alternatively, it may be that the overnight contact of chimpanzees with their beds is sufficient to allow body-associated organisms to accumulate, much as is the case for our own modern beds. To test these contrasting hypotheses, we sampled chimpanzee beds in the Issa Valley, western Tanzania.

## 2. Material and methods

The Issa valley is situated within the Greater Mahale Ecosystem in Tanzania. It is more than 90 km northeast from the nearest national park boundary (Mahale Mountains), and roughly 60 km southeast from the nearest town (Uvinza). This region is characterized by broad valleys, separated by steep mountains and flat plateaus, ranging from 900–1800 m above sea level. Vegetation is dominated by miombo woodland—*Brachystegia* and *Julbernardia* (Fabaceae), interspersed with swamp and grassland. A small proportion of the landscape (approximately 7%) is composed of evergreen gallery and thicket riverine forests. There are two distinct seasons: wet (November–April) and dry (May–October). Rainfall averages about 1200 mm per annum (range: 900–1400 mm, from 2001–2003; 2009–2014), and temperatures range from 11°C to 35°C [23,37]. The core study area (85 km^2^) is used by one community of chimpanzees. As chimpanzees in Issa are unhabituated to observers, the exact number of individual builders represented is unknown; however, previous work by Rudicell *et al*. [38] estimated this community to include approximately 67 individuals.

Within the study area, we collected microbes from chimpanzee beds (*n* = 41) and from environmental locations (*n* = 41), as well as the arthropods associated with a subset of those beds (*n* = 15 beds and 15 forest floor locations). Samples were collected between August 2013 and April 2014. All chimpanzee beds were sampled following abandonment. Bed age was calculated as time since construction and grouped into one of three classes; fresh = 1 day, recent = 2–7 days and old = 11–35 days (following Plumptre & Reynolds, [39]). Because the beds in our study were not used for more than one night, time since abandonment and bed age are the same. Additionally, though we know the identity of the chimpanzee community, we could not directly observe which chimpanzee used a given bed; therefore, we do not consider how individual variation influences the bacteria and arthropods present. We focus instead on the overall differences in how organisms in chimpanzee beds vary relative to the natural habitat. Fieldwork was approved by the Tanzanian Wildlife Research Institute (TAWRI) and the Commission for Science and Technology (COSTECH); permit no. 2014-202-ER-2011-94.

### 2.1. Microbial collection, processing and analyses

Dust samples to be used in microbial analyses were collected using dual-tipped sterile BBL™ CultureSwabs™, identical to those used to study homes in the USA [36,40], as well as the International Space Station [41]. We collected dust from two sample locations within each chimpanzee bed; a branch used for bed construction (*n* = 41 beds) and, for a subset of beds, a leaf that composed the mattress (*n* = 14 beds). As branches provide the structural support for chimpanzee beds, we would expect frequent contact during building, general activity and rest. Additionally, we collected two environmental samples from within the same tree, at a height similar to that of the sampled bed; a branch not incorporated into the bed (*n* = 41 locations) and a leaf not incorporated into the mattress (*n* = 14 locations). These paired, environmental sites would have presumably had much less exposure time, if any at all, to the chimpanzees. For our analyses, we pooled branch and leaf samples and considered differences in surface type as a potential explanatory factor in determining microbial diversity and community composition.

For each sample, we performed DNA extractions with a MO BIO PowerSoil® DNA Isolation Kit (12888-100). Under sterile conditions, we removed one swab and swirled it against the side of a PowerBead tube for 10 s. We conducted all subsequent microbial DNA extraction steps in accordance with the provided kit protocol, apart from step 19, in which we reduced the quantity of Solution C6 to 50 µl to concentrate the eluted DNA. We then sent extracted DNA to the Microbiome Core Facility, University of North Carolina Chapel Hill, School of Medicine (USA) for PCR amplification and sequencing on the Illumina MiSeq platform. We targeted an approximately 300 bp sequence, within the V1–V2 region of the 16S rRNA gene, with universal primers: 8F 5′-AGAGTTTGATCCTGGCTCAG-3′ and 338R 5′-GCTGCCTCCCGTAGGAGT-3′.

We merged overlapping reads with FLASH (v 1.2.11, [42]), set to allow a maximum overlap of 200 bp, and used the UPARSE pipeline (v 8.0.1623, [43]) to cluster sequences into operational taxonomic units (OTUs) at 97% similarity. We assigned taxonomy using the RDP Classifier 2.2 in QIIME [44,45], trained on the Greengenes database (v. 13_8, [46]), and identified a total of 8913 unique OTUs from 3 088 288 sequences. We removed low-quality or spurious OTUs by applying several filters to the dataset. OTUs were removed if they had a merged consensus sequence length outside the range of 310–370 bp, if they had less than 50 total reads across all samples, or if their taxonomy was flagged as cyanobacteria, mitochondria or unassigned (15% of total sequences; removed sequences in the electronic supplementary material, table S1). The filtered dataset contained 2 625 831 sequence reads over 1967 OTUs. We then rarefied those sequences to 5600 reads per sample and used the rarefied dataset for all downstream analyses. Of our 96 samples, four samples from within chimpanzee beds and four environmental samples did not meet the minimum rarefaction threshold. We analysed all data in the R environment with the *mctoolsr* and *vegan* packages [47–49].

Using our rarefied dataset, we compared differences in OTU richness (measured by the number of unique OTUs within a sample) and the Shannon diversity index among samples with Kruskal–Wallis tests. We tested the relative contribution of each potential explanatory factor on both OTU richness and microbial community composition with permutational multivariate analysis of variance (PERMANOVA), based on 999 permutations [50]. We quantified differences among microbial communities through square-root transformation and the Bray–Curtis dissimilarity metric and visualized community composition data with nonmetric multidimensional scaling (NMDS) ordination plots. We included all potential explanatory variables of interest within both the OTU richness and community composition PERMANOVA models, using the false discovery rate correction for multiple comparisons. Variables within these models included whether a sample was from a chimpanzee bed, the age of a bed, season (wet or dry), elevation above sea level (metres), and whether a sample was from a branch or a leaf.

To assess the extent to which the microbial community within chimpanzee beds is dominated by taxa from the same sources as those that are most abundant in human beds (i.e. faecal, skin and oral associates; [36]), we used a source-tracking approach similar to those used previously [36,51]. While the microbiota of humans and chimpanzees differ, a number of bacterial taxonomic groups are characteristically associated with mammals [52,53], and an even larger number is shared among great apes [54–56]. In order to determine whether a bacterial taxon is likely to have come from the faeces, skin or mouth of a chimpanzee, it would be ideal to characterize the microbes from the wild chimpanzees within our study sites. However, since this population of chimpanzees is unhabituated, we used body associate data from previous research. We used data collected from wild and sanctuary primate populations within Africa to define a list of bacterial taxa associated with chimpanzee faeces and mouths (faecal: [57–59]; oral: [60]; electronic supplementary material, table S2). Where data from wild chimpanzees were not available (i.e. skin associates), we used taxonomic groups defined from the skin samples of captive chimpanzees [61] augmented with bacterial taxa found by Ross [52] to be ubiquitous across mammal orders, including those of non-human primates (electronic supplementary material, table S2). We do so while acknowledging that some taxa common on the skin of wild chimpanzees might be missing in captive populations (as seen in faeces; [62–63]) and absent from other mammals. However, given the similarity of skin microbiomes across mammal orders [52], we think this to be a reasonable starting point. We tested all differences in the relative abundance of body-associated microbes between bed and environmental samples with Kruskal–Wallis tests.

### 2.2. Arthropod collection and analyses

We collected arthropod specimens from 15 chimpanzee beds, at two locations per bed, using a handheld insect vacuum (BioQuip products); inside the bed and the ground directly below the bed (*n* = 30). We vacuumed each bed and ground location for 2 min. After collecting samples, we stored them in 95% ethanol and shipped them to R.R.D.'s laboratory (NC State University) for specimen sorting and identification. M.A.B. identified arthropods to the lowest possible taxonomic rank, based on morphology from intact specimens, in the NC State Entomology and Plant Pathology laboratory. Owing to the great diversity of poorly characterized invertebrate species in Tanzania, particularly in the canopy [64], we were unable to identify many of the specimens to species, or even family, level. However, because the arthropods associated with primates have been well studied [65], we were confident that we could identify such specimens if present.

We calculated arthropod richness based on the identification of morphospecies and tested differences in abundance between chimpanzee beds and the ground directly below each bed with a Poisson distribution. We also assessed the likelihood of arthropods in the samples being chimpanzee bed or human home associates and calculated the total number of known or potentially blood-feeding ectoparasites based on biological information provided in the literature for the taxa recovered [36,65]. Here we did not consider how arthropod communities vary with bed age. We found so few ectoparasites that it was impossible to formally analyse differences among bed and forest floor locations or to quantify changes over time, beyond reporting our raw counts and the identification of each of the collected specimens.

## 3. Results

### 3.1. Microbes

We identified a total of 1896 microbial OTUs in chimpanzee beds and 1784 microbial OTUs from environmental samples. Proteobacteria, Actinobacteria and Bacteroidetes were the most common phyla, accounting for 92.4% of sequence reads from beds and 91.4% of sequence reads from environmental samples, with the phyla Proteobacteria and Actinobacteria accounting for nearly all OTUs present. The most common families of bacteria in both the chimpanzee beds and the surrounding environment were Methylocystaceae, Pseudonocardiaceae and Microbacteriaceae.

**Figure 1.**
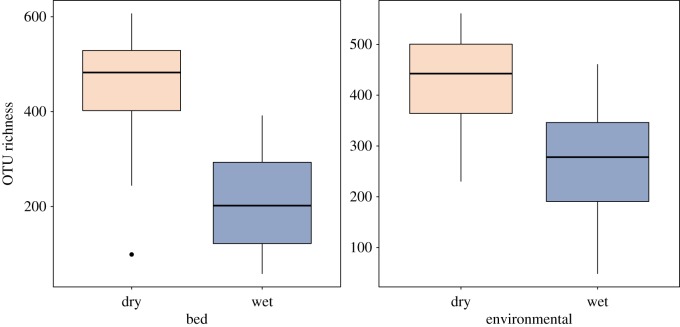
The OTU richness among all samples was primarily driven by differences in wet and dry seasons (*p* < 0.001). Season accounted for approximately 43% of the observed variation, with no difference between chimpanzee beds and the environment (*p* = 0.509). OTU richness was greatest in the dry season overall, as well as when chimpanzee beds or environmental samples were considered on their own (*R*^2^ = 0.54, *p* < 0.001; *R*^2^ = 0.32, *p* < 0.001, respectively).

We observed no differences in the OTU richness or Shannon diversity of microbes in chimpanzee beds, when compared to branches and leaves of the same tree (richness: *χ*^2^ = 0.071, *p* = 0.789; average OTU richness per sample: bed = 343, tree branch or leaf = 357; Shannon diversity: *χ*^2^ = 1.288, *p* = 0.256). When considering the relative contribution of all factors, season was the strongest determinate of OTU richness across all samples. Whether samples were collected in the wet or dry season accounted for nearly half of the observed variation (*R*^2^ = 0.43, *p* < 0.001), where richness was greatest during the dry season (figure 1). Elevation above sea level was the next most explanatory variable (*R*^2^ = 0.31, *p* = 0.011). When considering only the microbes found in chimpanzee beds, age of the bed and whether samples were taken from branches or leaves did not affect OTU richness (*p* = 0.631, *p* = 0.811, respectively; electronic supplementary material, table S3*a*).

Just as with OTU richness, differences in community composition among all samples was strongly influenced by season (*p* < 0.001) and elevation above sea level (*p* < 0.001). However, here elevation explained 46% of the total observed variation, whereas season accounted for only 13% (*p* < 0.001). Within beds, the presence of one or more chimpanzees was a determinate of microbial community composition, though the effect was small relative to the other factors (*R*^2^ = 0.03, *p* < 0.001; electronic supplementary material, figure S1). Bed age was not predictive of community assemblage (*p* = 0.714; electronic supplementary material, table S3*b*).

Of the top five most abundant bacterial genera known to be associated with chimpanzee faeces (as found in Yildirim *et al*. [58]), *Oscillabacter*, *Roseburia*, *Faecalibacterium* and *Caprococcus* were not found in any of our samples, regardless of whether the sample was collected in or outside of a chimpanzee bed. Even closely related genera in the *Oscillabacter* family, Oscillospiraceae, were not present. Faecal bacteria from the *Ruminococcus* genus were present but rare (occurred in just 5% of samples and accounted for 0.008% of sequence reads) and were no more abundant in beds than from environmental locations (*χ*^2^ = 2.857, *p* = 0.090). Even when we expanded our dataset to include all faecal taxa [57–59]; electronic supplementary material, table S2), we found no difference in the proportion of faecal bacteria present in beds relative to branches or leaves of the same tree (*χ*^2^ = 1.649, *p* = 0.199). Similar to the case for faeces, skin-associated bacteria were no more common in chimpanzee beds (*χ*^2^ = 0.154, *p* = 0.695; 2.4% of total reads) than in environmental samples. Particularly noteworthy was that, although *Corynebacterium* is the most abundant skin-associated taxonomic group currently described from chimpanzees (as well as from gorillas) [61], we found no *Corynebacterium* in chimpanzee beds. Oral bacteria, on the other hand, were more abundant in chimpanzee beds than on adjacent branches and leaves (*χ*^2^ = 14.644, *p* < 0.001). However, these too represented a very small portion of the total abundance of all microbes (0.82% of sequence reads from beds, 0.03% of sequence reads from the environment). Collectively, body-associated taxa (be they faecal, skin or oral in origin) accounted for only 3.5% of all microbial sequence reads from within chimpanzee beds.

### 3.2. Arthropods

Arthropods were more abundant on the ground than in chimpanzee beds (*p* = 0.007; *n* = 226 ground specimens, *n* = 108 bed specimens; table 1). Nonetheless, beds (*n* = 15) were host to 12 orders of arthropods, comprised 47 total morphospecies, with an average of 5.2 orders and 3.1 morphospecies represented per individual bed. Of all morphospecies collected just two are known ectoparasites of mammals (Phlebotominae and Ceratopogonidae, *n* = 3). All three specimens from these families were collected from within beds. We also collected one specimen of a potential blood-feeder from the Anthocoridae family (*n* = 1; table 1). We collected one Ceratopogonidae larva from the ground below a chimpanzee bed; however, though the adults of Ceratopogonidae are blood-feeders, the larvae are not, so this specimen was not included in the total number of ectoparasites.

**Table 1.** Arthropod specimens. (Specimens were identified to the family or group level. Presence/absence data were noted for chimpanzee bed and ground samples. All specimens indicated as parasites are from taxa that include ectoparasites.)

class	order	family or group	nest	ground	notes
Arachnida	Sarcoptiformes	Oribatida	X	X	single specimen in nest
		Astigmata		X	
	Trombidiformes	Erythraeidae		X	
		Bdelloidea		X	
	Mesostigmata	unidentified		X	
	unidentified ‘Acari’	unidentified		X	
	Araneae	Oonopidae	X		
		Oxyopidae		X	
		Salticidae		X	
		Selenopidae	X		
		unidentified	X	X	
	Pseudoscorpionida	unidentified		X	
Diplopoda	Polyxenida	unidentified	X	X	
Insecta	Collembola^a^	Entomobryidae	X	X	
		Symphypleona		X	
		unidentified	X	X	
	Zygentoma	Lepismatidae	X		
	Isoptera	unidentified		X	
	Orthoptera	Mogoplistidae	X	X	
		Tettigoniidae	X		
		unidentified	X	X	
	Blattodea	Ectobiidae		X	
	Hemiptera	Aphididae		X	
		Blissidae	X		
		Cicadellidae	X	X	
		Coccoidea	X		
		Dipsocoridae		X	
		Fulgoroidea	X	X	
		Lasiochilidae	X		
		Psylloidea	X	X	
		Reduviidae	X	X	
		Rhyparochromidae		X	
		Veliidae		X	*Hebrovelia*
		other Anthocoroidea^a^	X		
		other Auchenorrhyncha	X		
		unidentified	X	X	
	Thysanoptera	Phlaeothripidae	X	X	
		unidentified Terebrantia		X	
	Psocodea	unidentified	X	X	
	Hymenoptera	Agaonidae		X	
		Apidae		X	
		Braconidae	X		
		Eulophidae	X		
		Formicidae: Dolichoderinae	X	X	
		Formicidae: Formicinae	X	X	*Camponotus, Polyrhachis*
		Formicidae: Myrmicinae	X	X	*Crematogaster, Cataulacus, Monomorium, Strumigenys*
		Formicidae: unidentified	X	X	
		Platygastridae s.l.	X	X	includes Scelionidae
		Pteromalidae		X	
	Coleoptera	Carabidae	X	X	
		Chrysomelidae	X	X	Alticini
		Curculionidae	X		including Scolytinae
		Lycidae		X	larvae only
		Silvanidae	X		*Airaphilus*; found in four different nests
		Staphylinidae		X	
		Tenebrionoidea		X	
		unidentified		X	
	Diptera	Cecidomyiidae	X	X	
		Ceratopogonidae^a^	X	X	larva from ground; adults from nest; larva and one adult in Forcipomyiinae
		Chironomidae		X	
		Chloropidae	X		
		Drosophilidae	X		
		Hybotidae		X	
		Phoridae		X	
		Psychodidae^b^	X		Phlebotominae
		Sciaridae	X	X	
		Tipulidae		X	
		unidentified		X	
	Lepidoptera	unidentified	X	X	single unidentified moth from nest

^a^Denotes taxa that potentially feed on blood (ectoparasites).

^b^Denotes taxa that feed on blood (ectoparasites).

Of all arthropods collected within beds, none was from a lineage known to be strongly dependent on chimpanzees or mammal structures [65,66]. One potential exception was that of the silvanid beetles (Silvanidae). These beetles are often found in human homes [66]; however, after further identification, we found that the silvanid beetles collected from chimpanzee beds belonged to the genus *Airaphilus*. The beetles within this genus feed on fungal spores and dead plant material and are commonly found beneath the bark of dead trees or in leaf litter. Owing to their ecological niche, it is unlikely to be a group directly associated with chimpanzee bodies or structures ([67], Dr M. C. Thomas 2016, personal communication).

## 4. Discussion

The exposure of a mammal to pathogens, environmental bacteria, insects and other sympatric taxa is likely to be strongly influenced by the ecology of its sleeping place. We hypothesize that this has been the case for tens of millions of years, such that mammalian immune systems have evolved in the context of frequent exposure to environmental species. It has often been suggested that we have reduced the diversity of our exposures, as we have begun to spend more time indoors. Yet, though it has become increasingly clear that which species mammals, including humans, are exposed to can have both beneficial and detrimental effects on health and well-being, little is known about what those interactions might have been historically, or how such interactions vary among our living relatives. Here we present, to our knowledge, the first study of the organisms found in the sleeping place of a non-human mammal, that of wild chimpanzees.

Based on the study of human homes [36], one might hypothesize that the microbes found in chimpanzee beds would be less diverse than that of the adjacent environment, and further, that chimpanzee beds would be primarily composed of body associates. Instead, we found that the diversity of bacteria in chimpanzee beds was similar to that of the surrounding environment (electronic supplementary material, table S3*a*). In addition, taxa from chimpanzee bodies were almost entirely lacking in beds. Though we recognize that there is still more research needed on the characterization of microbiomes from wild chimpanzees, the near complete absence of currently defined body-associated taxonomic groups from within chimpanzee beds indicates that there is likely to be little accumulation of such species. The construction and likely inhabitation of a bed influenced which bacteria were present; however, the season in which each bed was built and the elevation above sea level explained most of the variation in microbial diversity and community assemblage (electronic supplementary material, table S3). Similarly, we found only four arthropod individuals known to be ectoparasites within beds, none of which appears to be a specialist on chimpanzees or their structures (table 1). In short, our results suggest that the microbes and arthropods to which chimpanzees are exposed while resting are predominately environmental, contingent upon season and location on the landscape.

The beds made by great apes, be they chimpanzees, gorillas, bonobos or orangutans, are typically used for a single night and then abandoned [22]. This movement of beds from one night to the next has long been thought to serve a range of beneficial functions. One explanation for such movement is that it decreases the ability of pathogens and pests to build up at a sleeping site and reduces the microbial odours associated with the individual that might attract predators [68,69]. Our results are commensurate with this hypothesis, as we found little evidence of the accumulation of bacteria or arthropods in chimpanzee beds. The lack of faecal bacteria may also be owing to chimpanzee toilette hygiene. Chimpanzees usually defecate over the sides of their beds [70]. Our data suggest they are effective at doing so in a way that prevents soiling the beds themselves. In addition, we found no arthropods in beds that were closely associated with chimpanzees and only four mobile blood-feeder specimens. Yet, chimpanzees are host to more than 60 parasites and pathogens, including lice and fur mites [65,71,72]. Given this, our results may reflect effective grooming practices (such as consuming ectoparasites), which prevent those species from reaching high abundances even when present. These findings highlight the need for more research on wild, habituated primate populations which would allow for the direct collection of microbes and arthropods from individuals and access to beds immediately following abandonment. We could then more fully explore the strength of individual variation, as well as directly observe behaviour within beds, which was not possible within the scope of our study.

### 4.1. Invention of the indoors

Though chimpanzees are not human ancestors, having diverged from a common ancestor between 6.6 and 12 million years ago [73,74], the building of beds by great apes is an ancestral trait that is thought to have appeared before the divergence of the hominid and hominin lineages [21–22,24]. Chimpanzees have often served as a model for reconstructing the behaviour of early hominin species [75–79], including the evolution of structure building [24]. Furthermore, it has been hypothesized that early hominins built beds in which to rest, as is seen among modern great apes [79–82]. Based on the reconstructed history of building among these groups, the beds of chimpanzees are likely to share common features with those of our hominin ancestors, especially given that our ancestors exhibited morphological adaptations for arboreality (*Ardipithecus ramidus*, [83]; *Australopithecus afarensis*, [84]; *Homo habilis*, [85]) and may have moved from sleeping site to sleeping site, as has been argued [37,81]. In as much, chimpanzee beds offer a window into the potential exposures of our ancestors while sleeping, even if an imperfect one.

Chimpanzee beds and human homes share two of the three most abundant microbial phyla (Proteobacteria and Actinobacteria). However, this similarity hides major differences in the likely origins of these microbes, differences that can be better seen if we consider the taxonomic level of families. Methylocystaceae, Pseudonocardiaceae and Microbacteriaceae were common in chimpanzee beds and are all previously described environmental microbes and/or soil associates [86–88]. By contrast, the most abundant families of bacteria in human homes are those associated with human skin or faeces; Streptococcaceae, Corynebacteriaceae and Lactobacillaceae [36]. To put it simply, we have created sleeping places in which our exposure to soil and other environmental microbes has all but disappeared, and we are instead surrounded by less diverse microbes that are primarily sourced from our own bodies [36,89]. The situation is similar with regard to arthropods. Chimpanzee beds contained no arthropod specimens specialized on life with chimpanzees. By contrast, the arthropod communities in human homes are diverse, often including hundreds of species, tens of which are specialized on life indoors with humans [6,66].

We do not yet know enough to reconstruct the complete history of human sleeping places and the species that composed their communities. However, we can propose based on our results from chimpanzee beds that at some point in hominin evolution, probably no earlier than a million years ago [90–91] and no later than 20 000 years ago [1–2], our ancestors made a major transition in terms of their exposures to other organisms while sleeping. They began to sleep repeatedly in the same spots and, in doing so, provided the opportunity for recurrent exposures to the subset of species that live on bodies and in beds and homes. With that change, the proportion of time we spend with these species has continued to increase, as we now spend the majority of our lives indoors. Meanwhile, our exposure to environmental microbes and arthropods has decreased. If true, exposure to our own microbes and to the arthropods adapted to the human built environment may be novel, relative not only to our recent history but also potentially to our more ancient past.

## Supplementary Material

Figure S1. Nonmetric multidimensional scaling (NMDS) ordination plot.

## Supplementary Material

Table S1. OTUs removed prior to rarefication and analyses.

## Supplementary Material

Table S2. Body associates.

## Supplementary Material

Table S3. PERMANOVA results.

## Data accessibility

Microbial data from this project were deposited in the Dryad data repository and made publically available at http://dx.doi.org/10.5061/dryad.7cp50 [92].
