# Supplementary figures and images for: Ecology of sleeping: the microbial and arthropod associates of chimpanzee beds
Source: R Soc Open Sci. 2018 May 16;5(5):180382. doi: 10.1098/rsos.180382 (PMC5990838; doi:10.1098/rsos.180382)

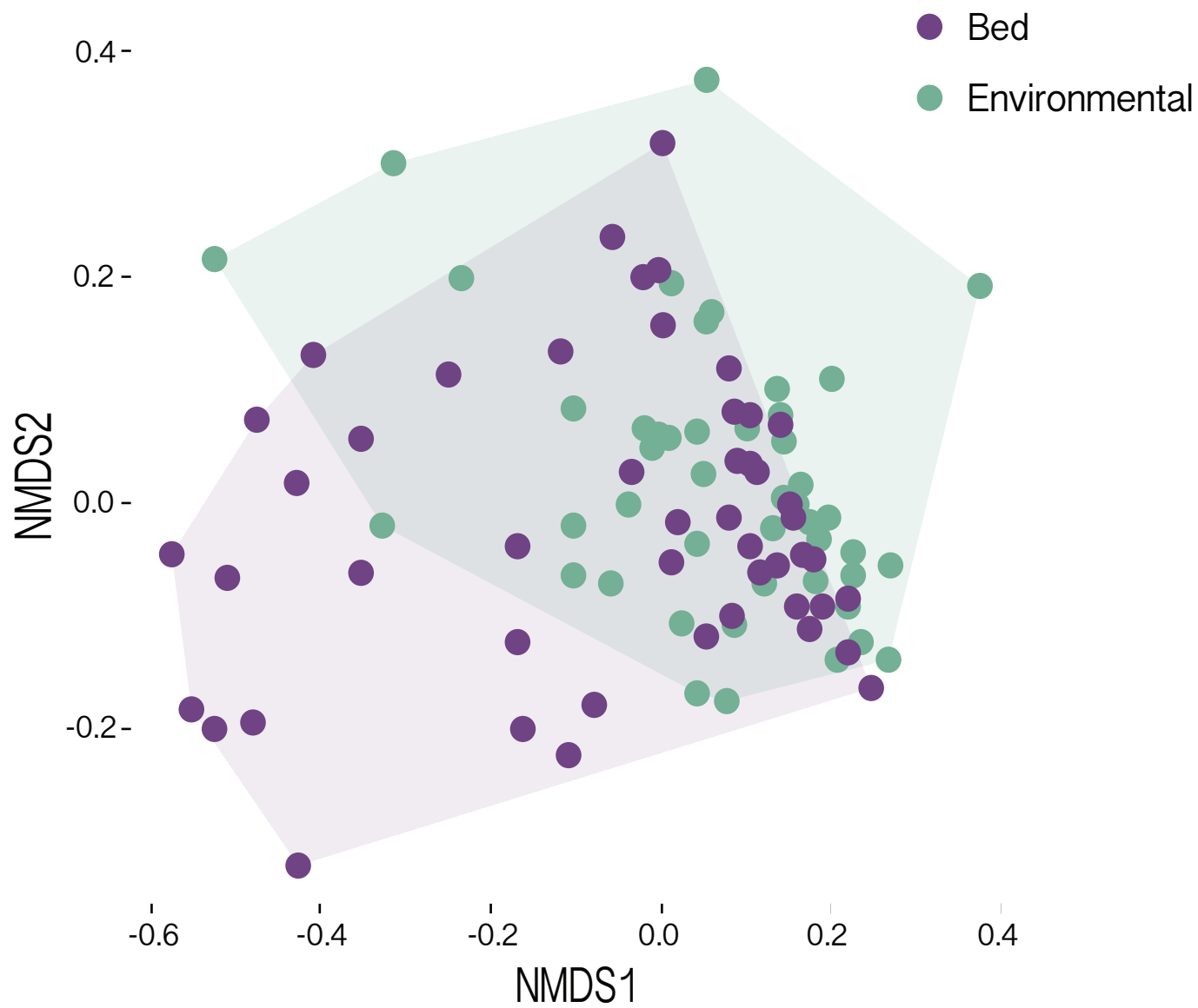

Supplement: Figure S1. Nonmetric multidimensional scaling (NMDS) ordination plot. [file rsos180382supp1.pdf]
